# Standardized testing and written communication improve patient understanding of beta-lactam allergy testing outcomes: A multicenter, prospective study

**DOI:** 10.1016/j.jacig.2022.05.003

**Published:** 2022-06-02

**Authors:** Jacqueline Loprete, Constance H. Katelaris, Louise Evans, Alisa Kane, Brendan McMullan, Brynn Wainstein, Melanie Wong, Jeffrey Post, Daniel Suan, Sanjay Swaminathan, Robyn Richardson, Jamie Rogers, Adrienne Torda, Dianne E. Campbell, Anthony D. Kelleher, Matthew Law, Andrew Carr, Winnie W.Y. Tong

**Affiliations:** a Immunology and HIV Unit, St Vincent’s Hospital, Sydney, Australia; b St Vincent’s Centre for Applied Medical Research, St Vincent’s Hospital, Sydney, Australia; c Campbelltown Hospital, Western Sydney University, Campbelltown, Australia; d School of Medicine, Western Sydney University, Campbelltown, Australia; e Liverpool Hospital, Liverpool, Australia; f Sydney Children’s Hospital, Randwick, Australia; g St Vincent’s Clinical Campus, School of Clinical Medicine, UNSW Medicine & Health, Sydney, Australia; h Children’s Hospital Westmead, Westmead, Australia; i School of Medicine, Sydney University, Sydney, Australia; j Prince of Wales Hospital, Randwick, Australia; k Westmead Hospital, Westmead, Australia; l Kirby Institute, University of New South Wales, Sydney, Australia

**Keywords:** Beta-lactam allergy, drug allergy, drug allergy testing

## Abstract

**Background:**

Historical penicillin allergy is commonly reported, but the lack of standardized allergy clinic practices may diminish the ability to delabel beta-lactam allergy appropriately.

**Objective:**

We sought to improve beta-lactam allergy testing and patient understanding of their antibiotic allergy status by standardizing testing and communication practices between 7 adult and pediatric hospital centers.

**Methods:**

Phase 1 prospectively described the beta-lactam allergy testing practices at each center. Following this, practice was standardized to achieve a defined panel of skin testing reagents, pro forma result letters for patients and referring doctors, and provision of medical alert jewelry to those with confirmed allergy. Testing outcomes and patient perception regarding allergy status 8 weeks postassessment were compared before (phase 1) and after standardization (phase 2). Primary outcomes were the percentage of participants delabeled after testing, and concordance rates between participant perception of their allergy status and their status as determined by the treating physician at 8-week follow-up.

**Results:**

Of 195 adult and pediatric participants (median age, 50 years; 21.5% <18 years; 36.9% males), 75% were delabeled of their beta-lactam allergy. No patient experienced anaphylaxis related to any beta-lactam delabeling testing. In phase 1, 75% of participants received written results, 52% were informed verbally, and 48% received results in more than 1 form. All phase 2 participants received written results (*P* < .01), 61% received verbal results from a physician as well (*P* > .05). At 8-week follow-up, 54% of phase 1 participants had concordant perceptions of their allergy status as the testing team versus 91.6% in phase2 (*P* < .001). Of the 17 participants who were delabeled and treated with a beta-lactam antibiotic during the 8-week follow-up period, there were no reported allergic reactions, although 1 participant experienced anaphylaxis following exposure to amoxicillin-clavulanic acid 1 year after delabeling.

**Conclusions:**

Standardization of testing and written patient information improved short-term patient perception of beta-lactam allergy status.

Eighteen to 25% of hospital inpatients report an antibiotic allergy.^1^^,^^2^ Such patients have longer hospital stays, more *Clostridium difficile*, methicillin-resistant *Staphylococcus aureus* and *vancomycin-resistant Enterococci* infections, and a greater chance of intensive care admission and in-hospital mortality, and incur higher costs than do patients without an antibiotic allergy label.^3^, ^4^, ^5^ Up to 54% of reported antibiotic allergies are to the beta-lactams, commonly referred to as “penicillin allergy.”^1^

Delabeling refers to the assessment and testing required to disprove a patient has an immune-mediated reaction to a medication. Antibiotic allergy delabeling can be time and resource-intensive, and requires dedicated challenge clinics and specialized health care staff trained in skin prick and intradermal testing techniques and interpretation and in the management of anaphylaxis. Although many expert consensus guidelines exist,^6^, ^7^, ^8^ there are no current standardized methods for assessing risk before proceeding to an antibiotic challenge, no standardized testing protocols, and no standards for communicating results to patients and other clinicians to ensure uptake of recommendations.

Antibiotic allergy testing generally results in 80% to 90% of patients being delabeled.^9^^,^^10^ However, a recent survey of Australian hospital specialists and pharmacists identified significant barriers to testing, including 42% of respondents who were either unaware of, or did not have access to, antibiotic allergy testing services.^11^ Furthermore, there are no published data to demonstrate effectiveness and long-term safety of allergy label removal. A retrospective study with telephone follow-up in Western Australia found that only 50% of delabeled patients were following posttesting recommendations, mainly due to ongoing patient or general practitioner reservations despite the negative testing.^12^

We hypothesized that implementation of an evidence-based, standardized protocol with accurate result dissemination for beta-lactam allergy delabeling would improve testing practices, the proportion of patients appropriately delabeled, and patient understanding 8 weeks after testing.

## Methods

### Participants

This study was performed at 6 hospital allergy services (Campbelltown Hospital, Children’s Hospital Westmead, Liverpool Hospital, St Vincent’s Hospital, Sydney Children’s Hospital, and Westmead Hospital) and an infectious disease service (Prince of Wales Hospital) providing pediatric and adult care in greater metropolitan Sydney.

Recruitment was open from May 2018 to February 2019 for phase 1, and September 2019 to December 2020 for phase 2 (this phase was longer due to COVID-19–related restrictions in outpatient visits). Patients were eligible if they were undergoing assessment for beta-lactam delabeling and they (or their parent/guardian) were able to provide written, informed consent. There were no exclusion criteria. The study protocol was approved by the South Western Sydney Local Health District Human Research Ethics Committee (reference HREC/17/LPOOL/490). The trial was registered with the Australian New Zealand Clinical Trials Registry (Trial ID ACTRN12618001386224).

### Study design

In phase 1, participants were assessed and followed as per existing local site protocols. Features of the index reaction of concern and additional medical and family medical history were recorded at the initial assessment. Assessment could include specific IgE testing, skin prick tests, intradermal tests, and provocation tests (antibiotic challenge) as directed by the treating clinician. The performance of specific IgE tests was at the discretion of the treating physician, and values greater than 0.35 kU/L were considered positive. Skin prick test results were considered positive if a wheal greater than 3 mm developed by 15 minutes following pricking. Histamine and glycerosaline were used as the controls. Skin prick test results were considered interpretable if the histamine response was greater than 3 mm and there was no response to the glycerosaline. Intradermal testing result was considered positive if there was a 3 mm increase in the size of the wheal at the injection site after 20 minutes. Intradermal testing with 0.9% saline was used as a negative control. Provocation tests were performed according to local protocols and clinician preference in regard to the antibiotic used, method of administration, the dose, and whether an extended provocation challenge was performed. Extended provocation tests consisted of continued administration of the antibiotic at home under the guidance of the treating physician. At sites where antibiotic allergy skin testing was not available (Prince of Wales), participants were assessed and challenged to 1 or more beta-lactams as clinically indicated.

Eight weeks following assessment, participants (or their parents/caregivers, if the participant was a child) were contacted via telephone to assess their understanding of their updated allergy status, to discern if they had taken any beta-lactam antibiotic since assessment was completed, and whether any adverse drug reactions had arisen from that administration.

Following phase 1, we used a Delphi method to survey the investigators to standardize testing and reporting procedures for phase 2. The following changes were implemented:

1. A minimum panel of reagents and concentrations for skin testing was instituted: benzylpenicilloyl poly-l-lysine (Diater, Madrid, Spain) 0.04 mg/mL, minor determinant mix (Diater) 0.5 mg/mL, ampicillin 20 mg/mL, and benzyl penicillin 6 mg/mL. Additional dilutions and reagents could be added at physician discretion (see Appendix 1 in this article’s Online Repository at www.jaci-global.org).
2. Measurement and recording of the initial bleb raised at the site of intradermal injection (p0) and of the wheal developed at the site 20 minutes following injection (p20).
3. All participants were verbally counseled regarding results by the supervising doctor on the day of testing.
4. A pro forma letter with these results and recommendations was mailed to each participant (or their caregiver) and mailed to their referring doctor and general practitioner (see Appendix 2 in this article’s Online Repository at www.jaci-global.org).
5. Updating of electronic medical records at time of testing and ensuring this information remains correct by time of 8-week follow-up.
6. All participants with allergy were supplied with medical alert jewelry free of charge.

Skin testing reagents could be added or omitted according to clinician discretion if this was felt to be appropriate in phase 2 (eg, limiting or omitting skin testing in children to reduce distress). The criteria for and performance of oral provocation tests continued to occur to local protocols and remained unchanged from phase 1.

Participants in phase 2 were again followed up via telephone 8 weeks after testing regarding their perceived allergy label and antibiotic prescribing.

### Outcome measures

The main outcomes were the proportion of participants who were delabeled at the end of testing, and the proportion of participants who had a perception of their allergy status that was consistent with the testing physician's at week-8 follow-up to identify the effectiveness of methods of dissemination of allergy assessment results.

### Statistical analysis

Initial recruitment for both phases combined was estimated as 150 participants over 12 months on the basis of previous patterns of clinical activity at each service. Initial phase 1 recruitment target was 75. Using these preliminary data, we calculated that to achieve improved correct perception in allergy status from 54% at follow-up in phase 1 to 80% in phase 2, recruitment of at least 95 patients in phase 2 would be required for a statistically powered study (alpha 0.05, power 0.8).

Data were analyzed using Stata 16.1 (Statacorp, College Station, Tex). A *Z* test was used to determine difference between demographic groups and phases. A chi-square test was used to assess association between history and index reaction and testing outcomes. A Cohen’s *K* was used to assess correlation between the outcome of testing and participant perception of allergy status at 8 weeks.

## Results

### Study participants and previous beta-lactam reactions

Characteristics of the 195 participants (85 in phase 1, 110 in phase 2) are described in Table I. We exceeded target recruitment in phase 2 because we had greater numbers present to clinics during this recruitment period. Participants in phase 2 were more likely to report having had an index reaction more than 10 years ago, and to be older than 18 years because the pediatric hospital allergy services were limited due to coronavirus disease 2019 pandemic during phase 2. Six participants recruited at Prince of Wales Hospital were not able to complete testing, because it was deemed that they required skin testing before direct provocation and this service was not available.

**Table I:** Demographic characteristics and index reaction

Characteristic	Phase 1 (N = 85)	Phase 2 (N = 110)	*P* value
Age (y), <18	31 (36.5)	11 (10)	<.00001
Sex, male	37 (43.5)	35 (31.8)	.09
Allergy to nonantibiotic drug	20 (23.5)	27 (24.5)	.87
History of asthma	20 (23.5)	23 (20.1)	.57
History of anaphylaxis to any cause	20 (23.5)	33 (30.0)	.31
Family history of antibiotic allergy	34 (40)	31 (28.2)	.08
Implicated antibiotic			
Penicillin			
All penicillins	68 (74.7)	74 (70.5)	.52
Amoxicillin	30 (32.9)	26 (24.8)	
Amoxicillin/clavulanic acid	2 (2.2)	11 (10.5)	
Unknown	27 (29.7)	35 (33.3)	
Other	9 (9.9)	2 (1.9)	
Cephalosporin	15 (16.5)	14 (13.3)	.53
Other	3 (3.3)	0	
Unknown	5 (5.5)	14 (13.3)	
Multiple antibiotics	1 (1.1)	1 (1)	
Features of reaction			
Mild/moderate immediate reaction	21 (24.1)	28 (25.5)	.83
Anaphylaxis	14 (16.1)	20 (18.2)	.70
Delayed hypersensitivity	29 (33.3)	40 (36.4)	.78
Other	8 (9.2)	8 (7.2)	.61
Unknown	15 (17.2)	14 (12.7)	.38
Time since reaction			
<1 mo	3 (3)	2 (1.8)	.58
1-6 mo	12 (14)	12 (10.9)	.40
6-12 mo	13 (15)	9 (8.2)	.13
1-5 y	17 (20)	18 (16.4)	.52
5-10 y	7 (8)	6 (5.5)	.48
>10 y	28 (31)	59 (53.6)	.001
Unknown	8 (9)	4 (3.6)	.1141

Values are n (%).

### Testing procedures and results

**Fig 1:**
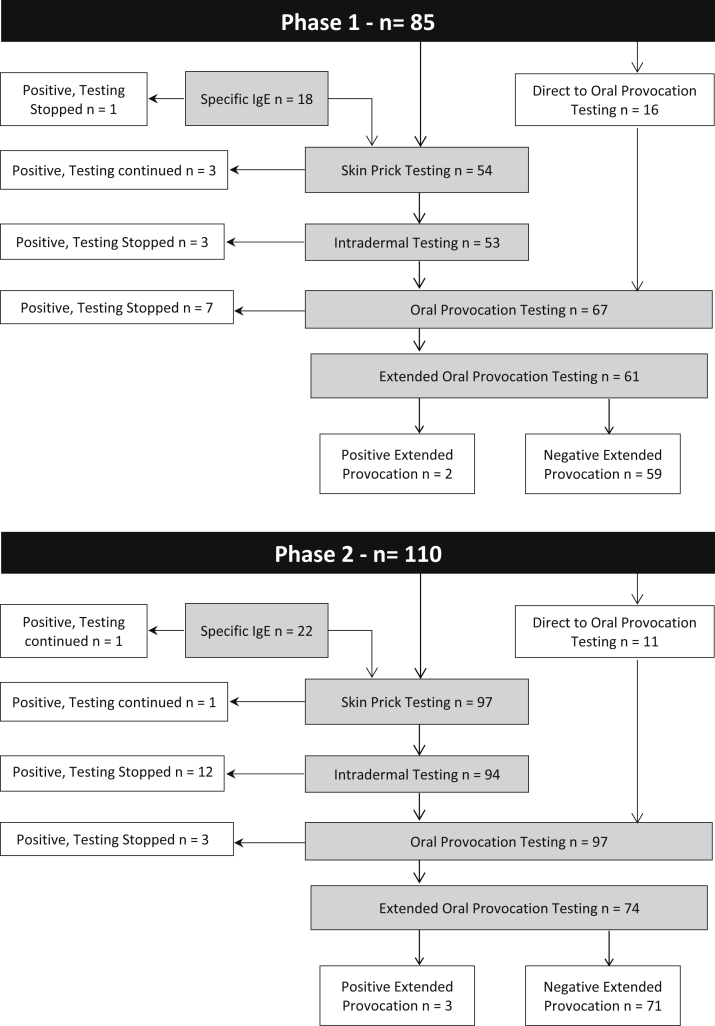
Participant flow through testing.

The flow of participant testing is described in Fig 1. Testing results are described in Table II. Drug-specific IgE was measured in 40 participants (20% overall, 21.2% phase 1, 20% phase 2; *P* > .05), with 2 positive results (5%). One went on to have further negative skin and provocation testing results, and both were assessed 6 to 12 months following their index reaction. Skin prick testing was performed in 151 participants (77.5% combined, 63.5% phase 1, 88.2% phase 2; *P* < .001), with 5 (3.3%) demonstrating positive test results. Of these 5 participants, 3 were assessed 1 to 6 months after their index reaction, and 2 were assessed more than 10 years after their index reaction. The most tested reagent was minor determinant mix (145 of 151), with a positivity rate of 0.7% (1 of 145). The skin prick test reagent with the highest positivity rate was cephalexin at 9% (1 of 11). Intradermal tests were performed in 147 participants (75.4% overall, 62.4% phase 1, 85.5% phase 2; *P* < .05), with positive results in 22 (15%). Eight of the positive participants were assessed more than 10 years after their index reaction, 6 within 1 to 5 years of their index reaction, 5 within 6 to 12 months of their index reaction, and 3 within 6 months. The most tested reagent was ampicillin (129 of 147 [87.8%]). The intradermal test reagent with the highest positivity rate was flucloxacillin (4 of 25 [16.0%]). There was no difference in the median number of skin test reagents used per participant between the phases.

**Table II:** Testing results

	Phase 1	Phase 2	Combined	*P* value
Specific IgE	1/18 (5.6%)	1/22 (4.5%)	2/40 (5.0%)	.88
Skin prick test	1/54 (1.9%)	4/97 (4.1%)	5/151 (3.3%)	.45
Intradermal test	9/53 (17.0%)	13/94 (13.8%)	22/147 (15.0%)	.74
In-clinic provocation test	7/74 (9.6%)	3/90 (3.4%)	10/164 (6.1%)	.11
Extended provocation test	2/61 (3.3%)	3/74 (4.1%)	5/135 (3.7%)	.81

Values are n positive/n tested (positive%).

The most common *in vivo* test across both phases was the oral provocation test (164 of 195 [84.1% overall]), with 10 (6.2%) yielding positive results. There was no significant difference in the use of oral provocation tests between the phases (78.8% phase 1, 88.2% phase 2; *P* > .05). The most common agent for oral provocation tests was amoxicillin (124 of 164 [76%]), with a positivity rate of 4.8% (6 of 124). The agent with the highest rate of positivity was cephalexin (6 of 14 [28.6%]). A further 3 provocation tests were performed intravenously with no positive results. Of the 10 positive challenges, 2 were in the same participant who reacted to amoxicillin and cephalexin. This participant had a mild to moderate reaction to amoxicillin 6 to 12 months before assessment. Of the other participants, 3 had a history of mild to moderate reactions to the index drug 1 to 6 months earlier, 4 had a history of a delayed reaction (3 more than 10 years earlier, one 1-6 months earlier), and 1 had a history of anaphylaxis more than 10 years ago. One participant who had a mild reaction to cephalexin at provocation test had not undergone skin testing.

A total of 135 participants (69.2%) underwent extended provocation challenges for 3 to 7 days (mean, 3.2 days). A total of 112 of these patients (82.9%) received oral amoxicillin, and 6 patients reported a reaction (5.4%, 5 mild, 1 moderate in severity). Four of these patients had an index history of delayed reaction to amoxicillin, 1 had anaphylaxis to cephalexin, and the index history of the remaining participant was unknown. The antibiotic with the highest rate of positivity was cephalexin (1 of 7 [14.3%]). There was no anaphylaxis as a result of in-clinic or extended provocation tests.

Participants were less likely to be delabeled if the index reaction was anaphylaxis (odds ratio, 0.69; *P* = .02) or if the participant had a history of anaphylaxis to any trigger (odds ratio, 0.34; *P* < .05). No other factors in regard to background history or index reaction were statistically significant in predicting outcome.

In phase 1, during which communication was not standardized, all participants received some form of communication regarding their testing results and amended or confirmed allergy status (Table III): 75% via a nonstandardized letter, and 52% were verbally informed of the results on the day of testing by either the supervising doctor or the nurse who administered the testing. About half the participants received communication in more than 1 form (48%); 31% of patients with allergy (5 of 16) in phase 1 were advised to obtain medical alert jewelry. In phase 2, 100% received standardized, written confirmation regarding their testing results and allergy status (*P* < .01 compared with phase 1) and 61% (67 of 110; *P* = .20) were recorded as also receiving results verbally from the physician, but results may have also been delivered verbally by nursing staff performing the testing; 59% of participants with allergy (20 of 34; *P* = .69) received a MediBand.

**Table III:** Communication of testing results

	Phase 1	Phase 2	*P* value
Verbal communication	44/85 (52%)	67/110 (61%)	.20
Written communication (letter via mail)	64/85 (75)	110/110 (100%)	<.01
Medical alert jewelry for allergic participants	5/16 (31%)	20/34 (59%)	.69

Values are n/N (%).

### Week-8 follow-up

Overall, 75% participants were delabeled (82% phase 1, 69% phase 2; *P* = .034).

At 8 weeks after testing, 160 (82.1%) participants were available for follow-up and asked what they considered their allergy status to be after testing. Only 35 (54%) of the participants in phase 1 had the same perception regarding their allergy status as the testing team (Table IV); 34% of delabeled participants considered themselves still allergic or were unsure regarding their status, and 81% of those who had their allergy confirmed considered themselves to be nonallergic (Cohen’s *K* of 0.13, poor agreement).

**Table IV:** Concordance between clinician diagnosis and participant perception 8 weeks after delabeling

		Participant perception		
Clinician diagnosis	Allergic	Nonallergic	Unsure	Total
*Phase 1*				
Allergy delabeled	13	32	4	49
Allergy confirmed	3	8	5	16
Total	16	40	9	65
*54% had concordant perception (Cohen’s K 0.13)*				
*Phase 2*				
Allergy delabeled	4	68	1	73
Allergy confirmed	19	0	3	22
Total	23	68	4	95
*92% had concordant perception (Cohen’s K 0.87)*				

In phase 2, concordant perception at week 8 increased significantly to 91.6% (Cohen’s *K* of 0.87; *P* < .001). There was also a significant reduction in the proportion of participants who were unsure of their status in phase 2 (13.8% vs 4.2%; *P* = .029). This improvement was seen in both the pediatric population (47.6% correct phase 1, 100% correct phase 2, Cohen’s *K* not applicable) and the adult population (79% correct [Cohen’s *K* = 0.15] phase 1, 90.5% correct [Cohen’s *K* = 0.87] phase 2) although this was significant in the pediatric population (47.6% vs 100%; *P* = .04) but not in adults (79.0% vs 90.5%; *P* = .78).

By the time of the week-8 follow-up, 37 (23.1%) participants had received antibiotics. Fifteen (40.5%) of these received a penicillin (6 in phase 1, 9 in phase 2). Of the 22 who received a nonpenicillin, 4 participants (18%, 2 in each phase) had been delabeled of their penicillin allergy. One participant who was delabeled in phase 2 to amoxicillin subsequently developed anaphylaxis following administration of amoxicillin-clavulanic acid 1 year after delabeling. The individual had a history of cephalexin-induced anaphylaxis 1 to 5 years before assessment. This participant was awaiting testing for clavulanic acid allergy and retesting to amoxicillin to delineate whether it is a *de novo* clavulanic acid allergy, a false delabeling to amoxicillin, or whether amoxicillin challenge acted as a sensitizing event.

## Discussion

Standardizing testing protocols and written communication of testing results improved participant understanding of their allergy status after beta-lactam delabeling from 54% concordance with the clinical team to 91.6%, and consequently may change future antibiotic prescribing.

The focus of studies looking at delabeling practices has long concentrated on methods of testing including skin testing regimens and provocation test practices. Few have reported how these practices translate beyond the allergy clinic or patient understanding after testing. Of 285 publications we identified looking at beta-lactam delabeling, only 3 addressed participant's understanding of the testing results after the fact.^12^, ^13^, ^14^ In these articles, 19.4% to 57.9% of delabeled participants considered themselves allergic to penicillin after testing. Previous studies have also looked at risk-stratifying patients so that testing is applied in a more streamlined manner. The focus on risk assessment and testing procedures is understandable given the known barriers to testing including limited facilities and how time intensive the procedure is for both clinician and patient.^15^ However, the importance of posttest care and communication may be underestimated.

When comparing the 2 phases of our study, more standardized testing regimes did not result in significantly different testing results. There were comparable rates of positivity across all forms of testing between the 2 phases. There was, however, a difference in the rate of delabeling. This may be partly attributed to the differences in age cohorts seen between the 2 phases (Table I). Recruitment in phase 2 was impacted by the coronavirus disease 2019 pandemic, and necessitated a longer than predicted recruitment period. During this time, hospital resources were redistributed and nonurgent patients were discouraged from attending appointments in person. At our sites, this resource change disproportionally affected our pediatric services compared with adult services. Although parent-reported rates of adverse reactions to penicillin range from 6% to 10% in children, reactions suggestive of IgE-mediated penicillin allergy account for only 1.16% of presentations for assessment,^16^ as compared with 5% of adults.^17^

Although we had higher rates of skin testing in phase 2 compared with phase 1, there was no difference in positivity rates. The negative predictive value of skin testing at predicting the outcome of a provocation test was high (Table IV), which agrees with current knowledge, but we were unable to assess the positive predictive value. This is because only a minority of participants who had positive skin test results went on to have provocation tests. Previous studies also demonstrate a low sensitivity of skin tests (30.7% in a meta-analysis of 105 articles^18^). To appropriately comment on the predictive value of tests, more studies need to be done where all participants undergo skin and provocation testing. However, our study does demonstrate that the additional benefit in adding the determinants to the negative predictive value compared with ampicillin and benzylpenicillin alone is small. Given the difficulty and expense associated with obtaining penicillin major and minor determinants, further work may demonstrate they are unnecessary for penicillin allergy assessment.

Further to this risk stratification, our study demonstrated that participants were less likely to be delabeled if the index reaction was anaphylaxis. In previous studies, the nature of the index reaction was also most predictive of outcome at testing.^19^, ^20^, ^21^, ^22^ Combining this risk stratification and robust data on the predictive values of skin tests may allow us to further streamline procedures, and possibly allow for direct provocation tests to be undertaken outside of allergy clinics, such as in primary care, which may be particularly helpful in regional and rural centers that lack allergy services.

Our study’s strengths lie in its multicentered recruitment of both adults and children, and in including both allergy and infectious disease services. The initial assessment of local practices allowed us to standardize practices across the differing sites. In addition, the benefits of improved communication demonstrated by this study could be translated to all drug delabeling, not just beta-lactams.

A limitation of this study is the relatively short follow-up period. We also had relatively low rates of subsequent beta-lactam use, likely reflective of this short follow-up period. There is also a need to address the reasons why individuals may continue to avoid penicillins after delabeling. A Western Australian study looking at 101 participants a median of 15 months following their allergy assessment found that 53.4% of participants had received an antibiotic, with 63.4% of those receiving a beta-lactam.^23^ Up to a quarter of their delabeled cohort were not following recommendations to prescribe beta-lactams. The most common reasons cited were a lack of trust in the testing and uncertainty on the part of the participants and their primary care providers. We need to assess our cohort to see whether these similar barriers exist, and whether the standardized communication in phase 2 may contribute to overcoming these barriers.

It would be useful to assess whether the standardization of testing and the risk stratification identified may allow for delabeling to occur outside of specialist allergy clinics, such as in general outpatient and general practitioner offices. This may represent a more cost-effective and accessible route for delabeling, increasing the appropriate use of beta-lactam antibiotics in the community.

We need to extend standardization in testing and written communication into nonpenicillin antibiotics and other drugs, particularly cephalosporins, to assess whether this practice improves posttesting outcomes for beta-lactam antibiotics alone or can be generalized for wider use. The knowledge base of skin testing to cephalosporins is smaller than penicillins, so a similar approach to one we applied here would be needed to fill this void.

With the emergence of multiresistant organisms and a dwindling antibiotic pipeline, simple, straightforward, and easily applicable testing protocols for penicillin and other beta-lactam allergy delabeling are needed. We must ensure that the results of delabeling continue beyond the day of testing through clear communication of results to both the participant and their primary care team.

**Key messages**

• **Beta-lactam allergy testing methodology is variable between centers, is resource and expertise-intensive and difficult to access, and patient understanding of the outcome of testing is inconsistent.**
• **Standardizing testing and written communication after beta-lactam allergy testing facilitated clearer messaging to patients and improved accuracy of understanding of testing outcomes.**
• **Results of beta-lactam allergy testing should be communicated to the patient in a written, standardized form.**

## Supplementary data

Appendices 1 and 2
