# Supplementary material for: Standardized testing and written communication improve patient understanding of beta-lactam allergy testing outcomes: A multicenter, prospective study
Source: J Allergy Clin Immunol Glob. 2022 Jun 2;1(3):99–105. doi: 10.1016/j.jacig.2022.05.003 (PMC10509847; doi:10.1016/j.jacig.2022.05.003)
Supplement: Appendices 1 and 2 [file mmc1.docx]

**Appendix 1: Standardised Skin Testing Protocol**

| **Drug** | **Reference concentration** | **Dilutions to prepare*** |
| --- | --- | --- |
| PPL | 0.04mg/mL | Neat (0.04mg/mL) |
| MDM | 0.5mg/mL | Neat (0.5mg/mL) |
| Benzylpenicillin | 600mg +2mLs=300mg/mL | Neat (6mg/mL) |
| Ampicillin | 1000mg +10mLs= 100mg/mL | Neat (20mg/mL) |

*Neat refers to highest non-irritant concentration and does **NOT** refer to reference concentration for penicillin testing.

| **Skin prick testing** | **wheal (mm x mm)** | **Notes** |
| --- | --- | --- |
| Negative control |  |  |
| Positive control (histamine) |  |  |
| Penicillin major determinant (PPL)  Neat (0.04mg/mL) |  |  |
| Penicillin minor determinant (MD)  Neat (0.5mg/mL) |  |  |
| Benzylpenicillin  Neat (6mg/mL) |  |  |
| Ampicillin  Neat (20mg/mL) |  |  |

| **Intradermal testing stage 1** | **Baseline** | **20-30 mins** | **Result** |
| --- | --- | --- | --- |
| Negative control (0.9% saline) |  |  |  |
| Penicillin major determinant (PPL)  1:10 (0.004mg/mL) |  |  |  |
| Penicillin minor determinant (MD)  1:10 (0.05mg/mL) |  |  |  |
| Benzylpenicillin  1:10 (0.6mg/mL) |  |  |  |
| Ampicillin  1:10 (2 mg/mL) |  |  |  |

| **Intradermal testing stage 2** | **Baseline** | **20-30 mins** | **Result** |
| --- | --- | --- | --- |
| Negative control (0.9% saline) |  |  |  |
| Penicillin major determinant (PPL)  Neat (0.04mg/mL) |  |  |  |
| Penicillin minor determinant (MD)  Neat (0.5mg/mL) |  |  |  |
| Benzylpenicillin  Neat (6mg/mL) |  |  |  |
| Ampicillin  Neat (20 mg/mL) |  |  |  |

**Appendix 2: Standardised Letter**

Site Specific Header Here

**IMPORTANT HEALTH INFORMATION**

Date: ………………………………….

**OFFICE USE:**

<Affix patient label here>

Dear Dr …………….…….,

………………………….. attended on the …./…./…. for beta-lactam antibiotic allergy testing to ……….. .

The history was of …*(describe reaction)*……………. following the use of ……(*state beta-lactam antibiotic)*……………..

In light of these results (Clinician to remove inappropriate statements):

- The patient IS NOT CONSIDERED ALLERGIC to__ *(insert beta-lactam antibiotic here)__* and related antibiotics, and this allergy label should be removed from all patient records
- The patient is considered ALLERGIC to __*(Beta lactam)__* but may be prescribed _*(other antibiotic)__* without specific precautions.
- The patient is considered ALLERGIC to __*(Beta lactam)*__and should also avoid __*(other antibiotic)* _ OR have it prescribed only with the following precautions ______________________.

🞏 A personalised medical alert / identification device has been given to the patient

🞏 If clinically indicated, desensitisation to this/these antibiotic(s) can be attempted under the supervision of a specialist immunologist/allergist, usually in a hospital setting.

Details of testing performed follow on page 2 of this letter.

If you have any queries, please contact the on-call Immunologist through………………………..

With kind regards,

Record of Testing:

Skin prick testing	Not performed 🞏

| Test drug / reagent | Result (Positive/Negative/Equivocal) |
| --- | --- |
| Negative control (glycerosaline) |  |
| Positive control (histamine) |  |
| Diater Major Determinant |  |
| Diater Minor Determinant Mix |  |
| Ampicillin 20mg/mL |  |
| Benzylpenicillin 6mg/mL |  |

Intradermal testing	Not performed 🞏

| Test drug / reagent | Result (Positive/Negative/Equivocal) |
| --- | --- |
| Negative control (normal saline) |  |
| Diater Major Determinant |  |
| Diater Minor Determinant Mix |  |
| Ampicillin 20mg/mL |  |
| Benzylpenicillin 6mg/mL |  |

In view of these results, the patient proceeded to a supervised, open challenge (provocation test) with:

Graded 🞏

| Antibiotic given………………max dose………… | Result |
| --- | --- |
| At 2 hours |  |
| Extended challenge (………… days) |  |
